# Sonophotochemical and photochemical efficiency of thiazole-containing metal phthalocyanines and their gold nanoconjugates

**DOI:** 10.55730/1300-0527.3596

**Published:** 2023-09-30

**Authors:** Nazli FARAJZADEH, Hacer Yasemin YENİLMEZ, Göknur YAŞA ATMACA, Ali ERDOĞMUŞ, Zehra ALTUNTAŞ BAYIR

**Affiliations:** 1 Department of Chemistry, İstanbul Technical University, Maslak, İstanbul, Turkiye; 2 Department of Chemistry, Yıldız Technical University, Esenler, İstanbul, Turkiye

**Keywords:** Phthalocyanines, gold nanoparticles, nanoconjugate, sonophotochemical, photochemical

## Abstract

This study presents the synthesis of some metal {M = Zn(II), Lu(III), Si(IV)} phthalocyanines bearing chlorine and 2-(4-methylthiazol-5-yl) ethoxy groups at peripheral or axial positions. The newly synthesized metal phthalocyanines were characterized by applying FT-IR, ^1^H NMR, mass, and UV-Vis spectroscopic approaches. Additionally, the surface of gold nanoparticles was modified with zinc(II) and silicon(IV) phthalocyanines. The resultant nanoconjugates were characterized using TEM images. Moreover, the effect of metal ions and position of substituent, and gold nanoparticles on the photochemical and sonophotochemical properties of the studied phthalocyanines was investigated. The highest singlet oxygen quantum yield was obtained for the lutetium phthalocyanine by applying photochemical and sonophotochemical methods. However, the linkage of the zinc(II) and silicon(IV) phthalocyanines to the surface of gold nanoparticles improved significantly their singlet oxygen generation capacities.

## 1. Introduction

Photodynamic therapy (PDT) is a well-known, modern cancer therapeutic approach that overcomes the drawbacks of traditional methods (e.g., chemotherapy, surgery, and radiotherapy). PDT mainly includes three elements: light, photosensitizer, and oxygen molecules. The photosensitizing agent is activated by exposure to light [1,2]. The interaction of the light-activated photosensitizer with oxygen molecules results in reverse oxygen species that can fight cancer lesions. However, poor penetration of light-activated sensitizers is a major disadvantage of this therapy [3,4]. Sonodynamic therapy (SDT) is a similar method, including an ultrasonic-activated sensitizer [5]. Although a sensitizer activated with an ultrasonic wave can penetrate deeper lesions, high values of sensitizer can be toxic to healthy cells [3,6]. Sonophotodynamic therapy (SPDT), originating from the combination of PDT and SDT, has attracted interest in recent years since this alternative method represents an efficient cancer treatment in which a low amount of photosensitizer can penetrate deeper lesions [3,7].

An appropriate photosensitizer should be nontoxic, inexpensive, pain-free, and selective to target cells. Additionally, it should be reliably activated by a certain light wavelength and easily removed from the patient’s body [3,6]. Although hematoporphyrin (Hp) and chlorophylls are extensively utilized for PDT applications, phthalocyanines have attracted significant attention owing to their excellent photosensitizing features [8–10]. Photosens is a sulfated aluminum phthalocyanine that has been reported to be a suitable photosensitizer for breast, gastrointestinal, lung, and skin malignancies as well as neovascularization of the eye. However, this compound exhibited neurotoxicity in rabbits during preclinical examinations [11]. Photocyanine is a mixture of di-(potassium sulfonate)-di-phthalimidomethyl zinc(II) phthalocyanine isomers that have been considered in phase II clinical studies since this complex displayed efficient phototoxicity against human hepatocellular carcinoma HepG2 [12].

Phthalocyanines are porphyrin analogs that contain an aromatic 18π-electron system. High electron transfers originating from the phthalocyanine ring result in unique chemical, physical, and optical properties [13–15]. Hence, the possibility of their use for various high-tech scientific applications has attracted interest in recent years. However, their utility is limited owing to poor solubility. π-Stacking interactions between the phthalocyanine rings lead to aggregation, which in turn results in low solubility/insolubility. The structural modification of the phthalocyanine ring periphery with long/bulky substituents and the insertion of metal ions into the phthalocyanine ring core enhance the distance between the rings, diminish aggregation, and improve solubility. Further, these changes refine the characteristic properties of phthalocyanines and allow the use of these compounds for certain applications [16–25]. For instance, the presence of axial ligand(s) in the structure of metal phthalocyanines shifts the electronic absorption spectra of the phthalocyanines to longer wavelengths and makes them appropriate agents for near-IR/IR applications [10,25]. Additionally, the nature of central metal ions significantly affects the photophysical, sonophotochemical, and photochemical properties of the phthalocyanines. In contrast to open-shell and paramagnetic metal-containing phthalocyanines, metal phthalocyanines that contain closed shells and diamagnetic ions, such as Zn^2+^, Al^3+^, Ga^3+^, and Si^4+^, exhibit high triplet quantum yield and long triplet lifetime, which are vital for suitable photosensitizing materials. Moreover, mono lutetium(III) phthalocyanines exhibit high photophysicochemical properties owing to the loss of the radical formed in the structure of their double-decker analogs. Therefore, closed-shell diamagnetic heavy metal-containing metal phthalocyanines are used as photosensitizing candidates for PDT and SPDT applications [26,27].

Heterocyclic materials are categorized as nitrogen-based, oxygen-based, or sulfur-based heterocyclic compounds. The nature of heteroatom, the ring size, and the substituent groups affect their physicochemical features. Since nitrogen-based heteroatom compounds are mostly found in nature and participate in the biological functions of animals and plants, they are included in the structure of low molecular weight drugs [28–30]. Azoles have a five-membered ring encompassing two hetero atoms (at least one is nitrogen) and are classified as nitrogen–oxygen (e.g., isoxazole, oxazole), nitrogen–sulfur (e.g., isothiazole, thiazole), and nitrogen–nitrogen (e.g., imidazole, tetrazole, triazole) heterocycles [31,32]. Among these materials, thiazoles have attracted significant attention in recent years. In particular, their potent utility in the design and development of new drugs for a wide range of biomedical applications including cancer has attracted the interest of pharmacists [33]. In addition, the presence of an electron-withdrawing atom like chlorine in the main system weakens the C=N bond, while an electron-donating group strengthens it. The specific cross-interaction effect of these substituents leads to a chemical shift of the carbon of the C=N bond. As the electron-withdrawing ability of the substituent increases, the energy gap becomes lower and in turn results in better biological, optical, and reactivity properties [34]. In the present study the aim was to design efficient PDT, SDT, and SPDT agents with all the biological characteristics of thiazole, chlorine, and phthalocyanines. Additionally, the modification of gold nanoparticles with the new agents can enhance their efficiencies.

Gold nanoparticles are well known as efficient metallic nanoparticles that have been utilized in a large number of cellular labeling, drug delivery, imaging, optoelectronic, and sensing applications since they exhibit high chemical stability and excellent optical features that originate from the higher surface area [35–38]. The low toxicity and biocompatibility of gold nanoparticles makes these metal nanostructures excellent alternatives for biological and therapeutic applications. Indeed, large numbers of biological materials and drugs can be placed on their surface owing to their high surface to volume ratio and small dimensions. The transport capacity of gold nanoparticles can be enhanced by modifying their surface. The surface of gold nanoparticles can be modified covalently (thiol or amino linkers) or noncovalently (electrostatic interactions) [39]. The components bound noncovalently to the surface of gold nanostructures become immediately therapeutically active after the delivery into the biological media compared to those covalently modifying the nanoparticles’ surface. The surface of gold nanostructures has been functionalized with diverse photoactive molecules [40–43]; however, in some studies the functionalization of their surface with phthalocyanines is reported [44–47]. As photosensitizers are mostly hydrophobic and tend to aggregate due to their planar heterocyclic structures, their low photoactivity can weaken PDT efficiency. Therefore, the placement of photosensitizing materials on the surface of gold nanoparticles leads to the preservation of their monomeric forms. Additionally, these surface modifications increase the solubility of the hydrophobic photosensitizers in aqueous media and make intravenous injections easy. Moreover, the hydrophobic photoactive molecule is required to penetrate the membrane of tumor cells [48]. Hence, the conjugation of phthalocyanines with gold nanoparticles can improve the PDT efficiency of phthalocyanines.

Our study can hopefully fill this gap in the literature. In the study, three metal phthalocyanines [M = Zn, Lu, Si] were synthesized. The resultant zinc(II) and silicon(IV) phthalocyanines were used for modification of the surface of gold nanoparticles. The singlet oxygen production abilities of the phthalocyanines and the gold nanoconjugates were studied using photochemical and sonophotochemical methods.

## 2. Experimental

### 2.1. Materials

Silicon(IV) phthalocyanine dichloride, lutetium(III) acetate, zinc(II) acetate, DBU, sodium hydride, hydrogentetrachloroaurate, 2-(4-methylthiazol-5-yl)ethanol, trisodium citrate, THF, chloroform, toluene, *n*-pentanol, *n*-hexane, and methanol were purchased from Merck. All the chemicals were used as received without any further purification. Compounds **1** and **2** were synthesized as described previously [49].

### 2.2. Synthesis of compound 3

Phthalonitrile derivative (**1**) (100 mg, 329 mmol) and lutetium(III) acetate (29 mg, 82 mmol) were dissolved in *n*-pentanol in the presence of the catalyzed amount of DBU at reflux temperature for 24 h. The content was cooled to room temperature and treated with a mixture of methanol and water (1:1). The precipitant was filtered off, washed several times with hot *n*-hexane, and dried in an oven. The product was purified using silica-gel column chromatography that was eluted with THF:chloroform (2:1). Yield: 38 mg (32%), mp > 250 °C. FT-IR: cm^−1^ 3099 (Ar–H), 2985-2880 (aliphatic C–H), 1690 (C=O), 1598 (C=C), 1555 (N=C), 1119 (C–O–C), 1038 (C–S–C). UV-Vis (DMSO): λ_max_/nm (logɛ) 346 (4.87), 693 (4.93). ^1^H NMR (500 MHz, d_6_-DMSO): δ/ppm 8.55 (s, 4H, S–CH–N), 7.43 (s, 4H, Ar-H), 7.04–7.01 (m, 4H, Ar–H), 4.73–4.71 (t, 4H, O–C**H**_2_–CH_2_), 4.58–4.54 (t, 4H, O–C**H**_2_–CH_2_), 4.22–4.20 (bs, 8H, O–CH_2_–C**H**_2_), 2.48 (s, 12H, CH_3_), 2.19–2.16 (s, 3H, acetate CH_3_). C_58_H_43_Cl_4_LuN_12_O_6_S_4_; MS m/z calcd. for [M]^+^ 1449.07 found 1419.50 [M–2Cl+K+2H]^+^.

### 2.3. Synthesis of compound 4

Silicon(IV) phthalocyanine dichloride (100 mg, 163 mmol), 2-(4-methylthiazol-5-yl)ethanol (23 mg, 163 mmol), and sodium hydride (12 mg, 491 mmol) were stirred in toluene (3 mL) at reflux temperature for 18 h under an inert atmosphere. After evaporation of the solvent, the pure product was obtained using column chromatography on alumina as the stationary phase and THF as the eluent. Yield: 46 mg (39%), mp > 250 °C. FT-IR: cm^−1^ 3093 (Ar–H), 2927–2866 (aliphatic C–H), 1642 (C=C), 1547 (N=C), 1051 (C–S–C), 952 (Si–O–C). UV-Vis (DMSO): λ_max_/nm (logɛ) 354 (4.52), 677 (4.92). ^1^H NMR (500 MHz, d_6_-DMSO): δ/ppm 9.44 (bs, 1H, S–CH–N), 8.15 (bs, 8H, Ar–H), 7.04 (bs, 8H, Ar–H), 2.23 (s, 3H, CH_3_), 1.54–1.52 (m, 2H, O–CH_2_–C**H**_2_), 0.91–0.89 (m, 2H, O–C**H**_2_–CH_2_). ^13^C NMR (500 MHz, d_6_-DMSO): δ/ppm 149.89, 149.53, 148.94, 135.68, 131.02, 128.38, 123.65, 62.16, 29.46, 14.66. C_38_H_24_ClN_9_OSSi; MS m/z calcd. for [M]^+^ 718.27 found 682.98 [M-Cl]^+^.

### 2.4. Preparation of nanoconjugates (G-2 and G-4)

The synthesis and surficial modification of gold nanoparticles were carried out as described in the literature [46,47]. Briefly, gold nanoparticles were prepared as described previously with some modifications [50]. Aqueous hydrogen tetrachloroaurate (1% w/v, 100 mL) was heated under reflux. Trisodium citrate solution (1% w/v, 5 mL) was added dropwise to the solution followed by vigorous stirring. The reaction was stopped when the yellow color of the solution changed to red and it was stored in a refrigerator. After addition of compound **2** or **4** (10 mg) dissolved in DMSO to the gold nanoparticles solution (9 mL), the mixture was vigorously stirred for 24 h at room temperature. Then the mixture was filtered off and dried.

### 2.5. Photophysical, photochemical, and sonophotochemical studies

The photophysical, photochemical, and sonophotochemical methods are explained extensively in the Supplementary section.

## 3. Results and discussion

### 3.1. Synthesis and characterization

**Scheme 1:**
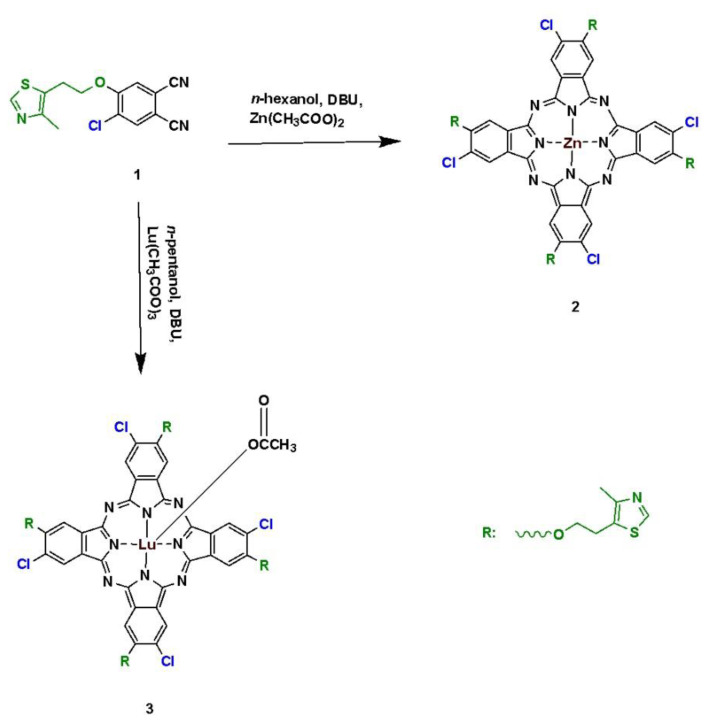
The synthetic pathways for compounds **2** and **3**.

**Scheme 2:**
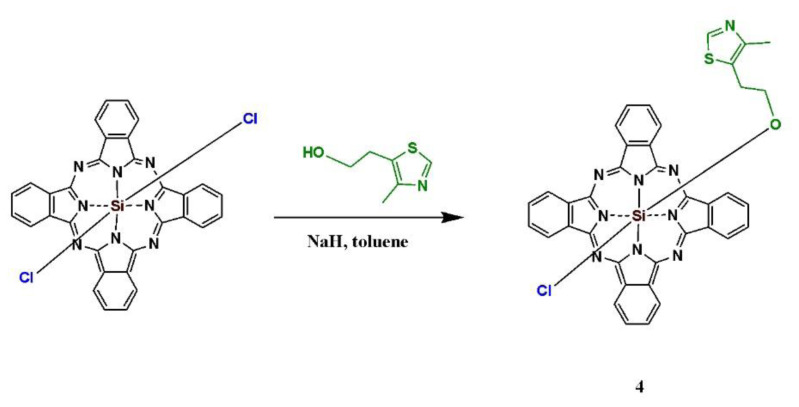
The synthetic pathways for compound **4**.

Compounds **1** and **2** were synthesized according to the methods applied in our previous study [49]. The synthetic route for macromolecule **3** is shown in Scheme 1. Compound **1** was cyclotetramerized in the presence of lutetium(III) acetate in *n*-pentanol at reflux temperature using DBU as the basic catalyst. The synthetic pathway for compound **4** is demonstrated in Scheme 2. Compound **4** was synthesized by the replacement of a chlorine atom with 2-(4-methylthiazol-5-yl) ethoxy through the nucleophilic substitution reaction. The reaction was carried out in toluene and catalyzed by sodium hydride. The newly synthesized compounds were characterized by performing FT-IR, ^1^H NMR, mass, and UV-Vis analysis. In the FT-IR spectrum of compound **3**, the respective vibration bands of aromatic and aliphatic C–H groups appeared at 3099 and (2985, 2880) cm^−1^, whereas the stretching vibrations of C=O, C=C, and C=N were observed at 1690, 1598, and 1555 cm^−1^, respectively. Additionally, the vibration bands of the C–O–C and C–S–C groups appeared at 1119 and 1038 cm^−1^, respectively. In the FT-IR spectrum of compound **4**, the respective vibration bands of the aromatic and aliphatic C–H groups appeared at 3093, and 2927 and 2866 cm^−1^, whereas the stretching vibrations of C=C and C=N were observed at 1642 and 1547 cm^−1^, respectively. The vibration bands of the C–S–C and Si–O–C groups appeared at 1051 and 952 cm^−1^, respectively. In the ^1^H NMR spectrum of compound **3**, the aromatic protons were observed at 8.55 (s, 4H, S–CH–N), 7.43 (s, 4H, Ar–H), and 7.04–7.01 (m, 4H, Ar–H) ppm, whereas the aliphatic ones appeared at 4.73–4.71 (t, 4H, O–C**H**_2_–CH_2_), 4.58–4.54 (t, 4H, O–C**H**_2_–CH_2_), 4.22–4.20 (bs, 8H, O–CH_2_–C**H**_2_), 2.48 (s, 12H, CH_3_), and 2.19–2.16 (s, 3H, acetate CH_3_) ppm. In the ^1^H NMR spectrum of compound **4**, the aromatic protons were observed at 9.44 (bs, 1H, S–CH–N), 8.15 (bs, 8H, Ar–H), and 7.04 (bs, 8H, Ar–H) ppm, while the aliphatic ones appeared at 2.23 (s, 3H, CH_3_), 1.54–1.52 (m, 2H, O–CH_2_–C**H**_2_), and 0.91–0.89 (m, 2H, O–C**H**_2_–CH_2_) ppm. In the ^13^C NMR spectrum of compound **4**, the aromatic protons were observed between 149.89 and 123.65 ppm, whereas the aliphatic ones appeared between 62.16 and 14.66 ppm. In the UV-Vis spectra of compounds **3** and **4**, the respective Q-bands were observed at 693 and 677 nm, whereas the B-bands appeared at 346 and 354 nm, respectively. The molecular ion peak of compound **3** appeared at 1419.50 m/z assigned to [M–2Cl+K+2H]^+^, whereas that of compound **4** was observed at 682.98 m/z corresponding to [M–Cl]^+^.

**Figure 1:**
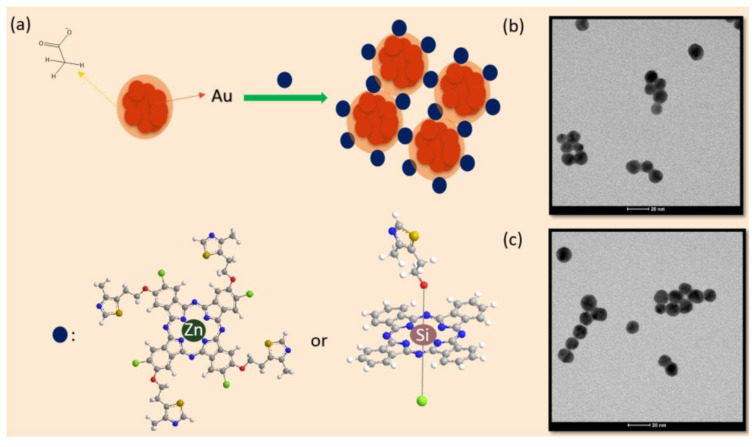
(a) Schematic representation of the conjugation of compounds **2** and **4** with gold nanoparticles; (b) the TEM image of nanoconjugate **G-2**; (c) the TEM image of nanoconjugate **G-4**.

Compounds **2** and **4** were conjugated with the surface of gold nanoparticles through the noncovalent interactions between N and/or S of thiazole groups and citrate groups of gold nanoparticles as well as π–π interactions (Figure 1a) [46,47,51,52]. The phthalocyanine-modified gold nanoparticles were characterized using TEM images (Figures 1b and 1c). The functionalization of the surface with phthalocyanines led to slight enlargement of gold nanoparticles. Moreover, the π–π interactions between the functionalizing phthalocyanines resulted in the approach of adjacent gold nanoparticles (larger single or twinned particles). Figure 1 shows the TEM images of nanoconjugates **G-2** and **G-4**. The size of the synthesized gold nanoparticles was 12–15 nm and increased slightly (approximately 2 nm) after the surface modification with compounds **2** and **4** (Figures 1b and 1c). The prepared gold nanoparticles and their conjugation properties were similar to those synthesized in our previous studies [46,47].

**Figure 2:**
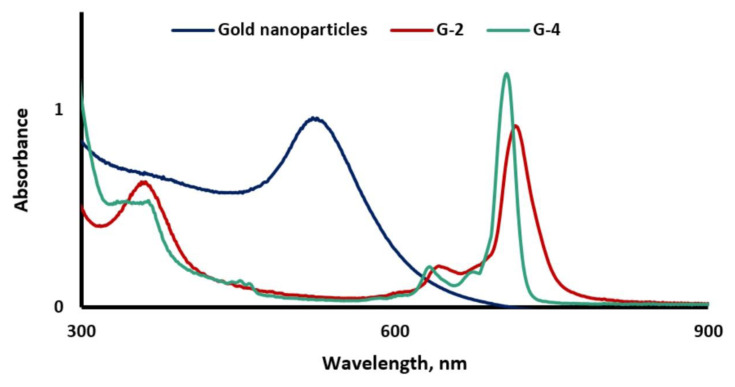
The UV-Vis spectra of gold nanoparticles and nanoconjugates **G-2** and **G-4**.

The UV-Vis spectra of the gold nanoparticles and nanoconjugates **G-2** and **G-4** are depicted in Figure 2. The surface plasmon resonance (SPR) absorption band of the gold nanoparticles observed at 517 nm disappeared after surficial functionalization with compounds **2** and **4** and confirmed the complete surficial modification. Further, the electronic absorption spectra of compounds **2** and **4** slightly blue-shifted approximately 3 nm in the presence of gold nanoparticles [53].

### 3.2. Fluorescence quantum yields (Φ*_F_*)

**Figure 3:**
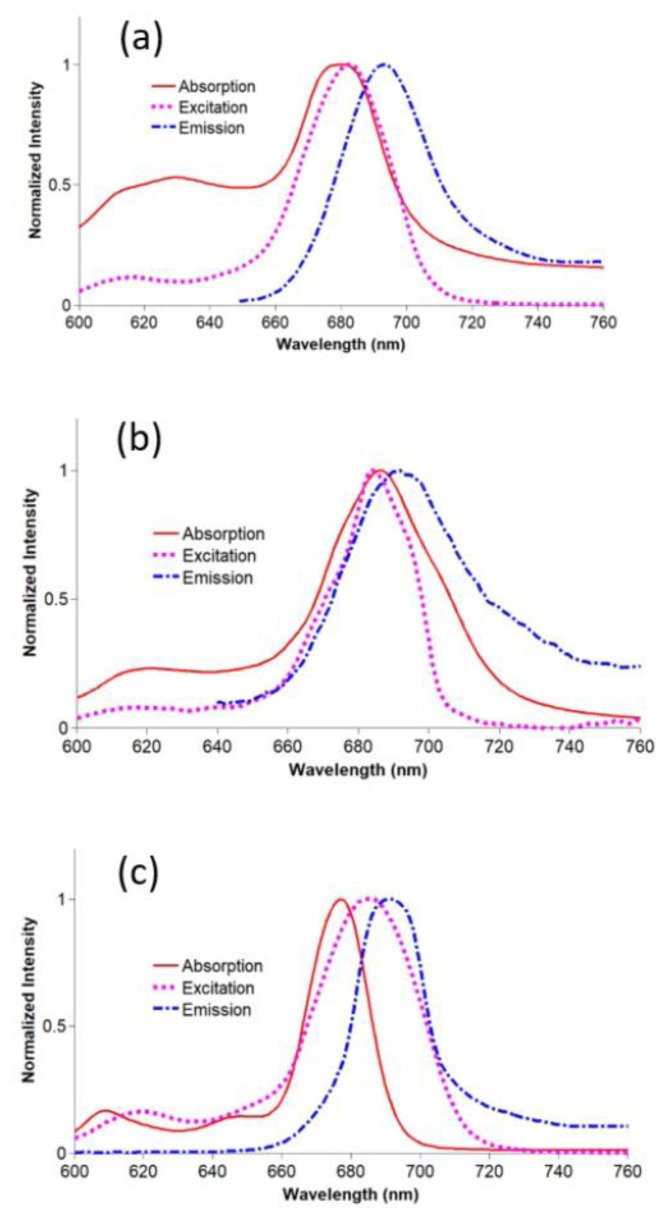
a) Absorption (679), excitation (681), and emission (692) spectra of **2** in DMSO; b) Absorption (677), excitation (683), and emission (690) spectra of **3** in DMSO; c) Absorption (686), excitation (684), and emission (692) spectra of **4** in DMSO (concentration: 6 × 10^−6^ M).

**Figure 4:**
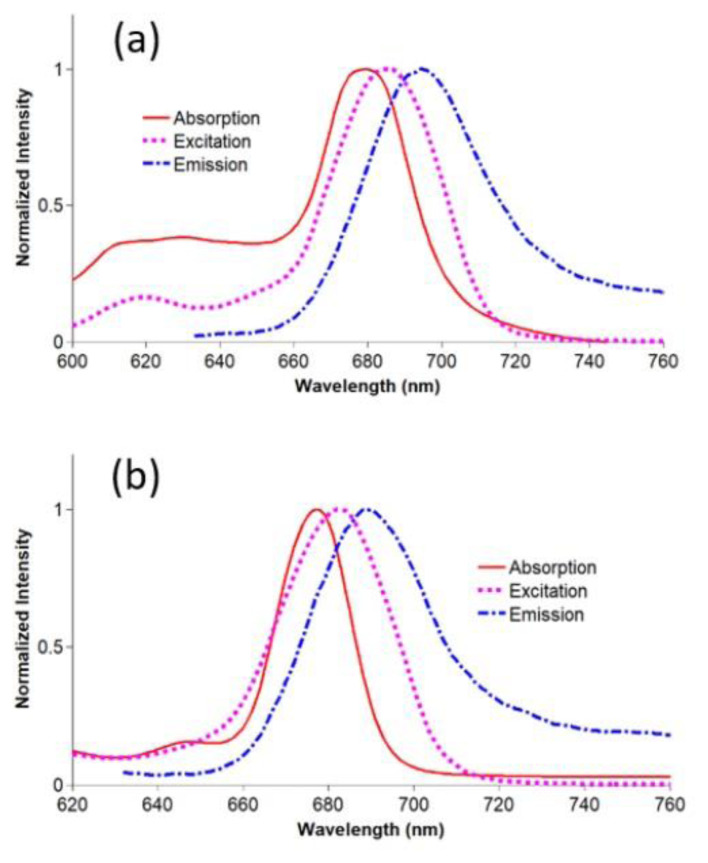
a) Absorption (679), excitation (681), and emission (692) spectra of **G-2** in DMSO; b) Absorption (686), excitation (684), and emission (692) spectra of **G-4** in DMSO (concentration: 6 × 10^−6^ M).

Fluorescence quantum yield is a measure to evaluate the absorbing photon energy efficiency of a sensitizer during phototherapy. The fluorescence properties of compounds **2**–**4** and gold nanoconjugates **G-2** and **G-4** were examined in DMSO. The spectral changes are shown in Figures 3 and 4. The fluorescence quantum yields are listed in the Table. The Φ_F_ values of nanoconjugates **G-2** and **G-4** were lower than those of compounds **2** and **3**. These findings confirmed the fluorescence quenching properties of gold nanoparticles [45]. Moreover, gold nanoparticles can promote intersystem crossing via the heavy atom effect [54]. As the *Φ_F_* values of unsubstituted ZnPc (Φ_F_ = 0.20 in DMSO) and SiPcCl_2_ (Φ_F_ = 0.44 in DMSO) were higher than those of compounds **2**–**4** and gold nanoconjugates **G-2** and **G-4** [49,50], the thiazole-based substituents can result in higher singlet oxygen quantum yields [55,56]. Nas et al. synthesized new tetrasubstituted metal-free and metal (Pb(II) and Zn(II)) phthalocyanines bearing benzothiazole and studied their photophysicochemical properties. The lowest fluorescence quantum yields were obtained between 0.138 (for lead(II) phthalocyanine) and 0.320 (for zinc(II) phthalocyanine) [57]. Peteni et al. studied the photophysical and photochemical properties of tris[(4-(pyridin-4-ylthio)-2-thio-4-methylthiazol-5-yl)acetic acid phthalocyaninato] zinc(II) and its silica nanohybrids (linked to and doped onto SiNPs). The highest quantum yield was obtained for the zinc(II) phthalocyanine (0.09) that decreased after chemical modification [58]. The quantum yields of compounds **2**–**4** and nanoconjugates **G-2** and **G-4** were in accordance with the literature.

### 3.3. Singlet oxygen quantum yield (Φ_Δ_)

**Figure 5:**
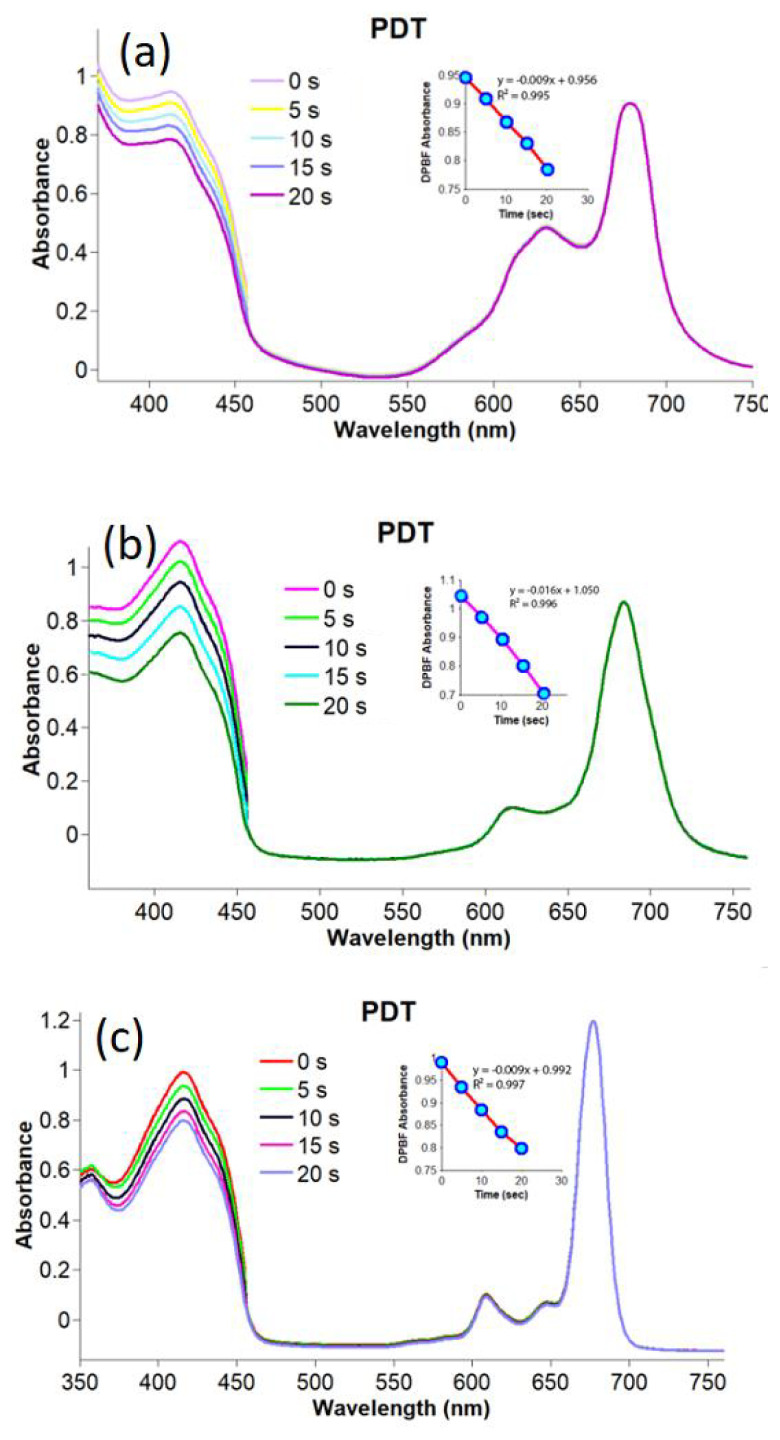
Representative absorption spectral changes during the determination of the singlet oxygen quantum yields for compounds **2**–**4** by photochemical applications for a) compound **2**, b) compound **3**, and c) compound **4** in DMSO by photochemical applications (concentration: 6 × 10^−6^ M).

**Figure 6:**
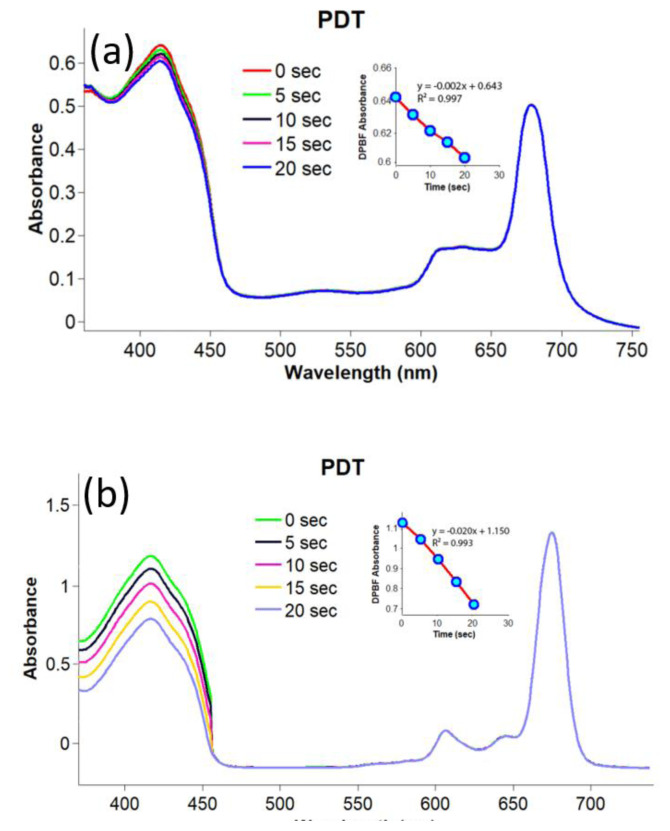
Representative absorption spectral changes during the determination of the singlet oxygen quantum yields for gold nanoconjugates compounds in DMSO by photochemical applications; a) nanoconjugate **G-2** (concentration: 4 × 10^−6^ M) and b) nanoconjugate **G-4** (concentration: 6 × 10^−6^ M).

Singlet oxygen is responsible for cytotoxic reactions in photodynamic/sonophotodynamic therapy. Therefore, the singlet oxygen generation capacity of a photosensitizing candidate is usually evaluated to measure its potency as an appropriate photosensitizer in these applications. In the present study, the singlet oxygen production ability of compounds **2**–**4** and gold nanoconjugates **G-2** and **G-4** was studied using photochemical and sonophotochemical methods. The singlet oxygen generation of compounds **2**–**4** and gold nanoconjugates **G-2** and **G-4** was determined by monitoring their UV-Vis absorption spectra in the presence of DPBF as a quencher. The related spectra are shown in Figures 5 and 6. The obtained Φ_Δ_ values are given in the Table.

The observation of no change in Q band intensities during sonophotochemical and photochemical studies confirmed that compounds **2**–**4** and gold nanoconjugates **G-2** and **G-4** were resistant to light and/or ultrasound. Due to the heavy metal effect of lutetium, the Φ_Δ_ value of compound **3** was higher than the Φ_Δ_ values of compounds **2** and **4** by applying photochemical methods. Additionally, the conjugation of compounds **2** and **4** with gold nanoparticles significantly improved the singlet oxygen production capacities of these metal phthalocyanines. Due to the heavy metal effect, metallic nanoparticles can increase the T1 population through the enhancement of spin-orbital coupling and, in turn, increase singlet oxygen generation [59,60]. These findings were in accordance with the literature reporting improvement in the Φ_Δ_ values of phthalocyanines after linkage to the surface of gold nanoparticles [61,62].

**Figure 7:**
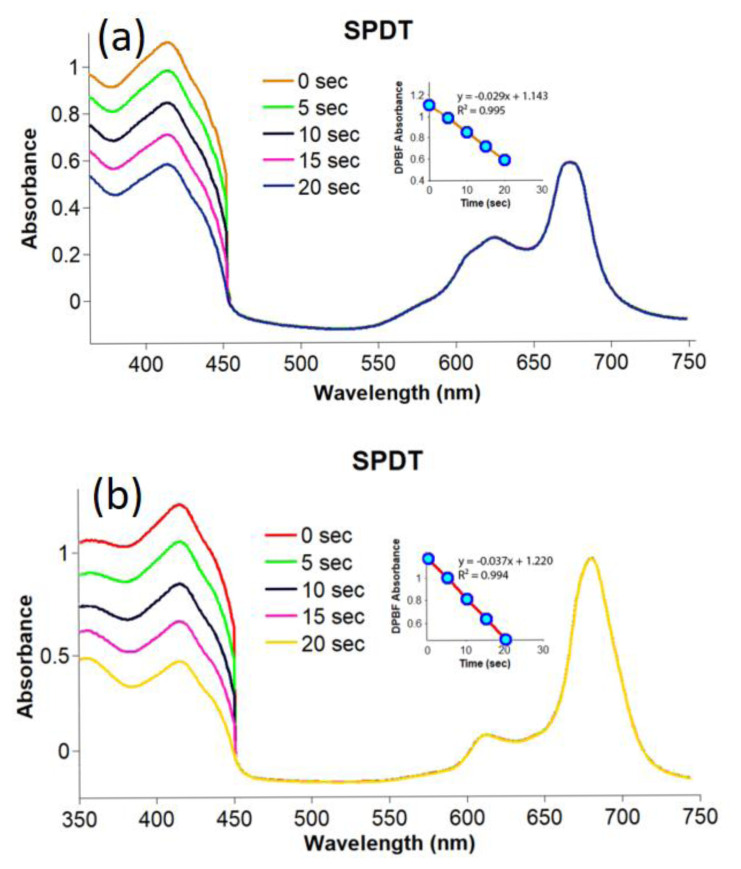
Representative absorption spectral changes during the determination of the singlet oxygen quantum yields for compounds **2**–**4** in DMSO by sonophotochemical applications; a) compound **2**, b) compound **3**, and c) compound **4** (concentration: 6 × 10^−6^ M).

**Figure 8:**
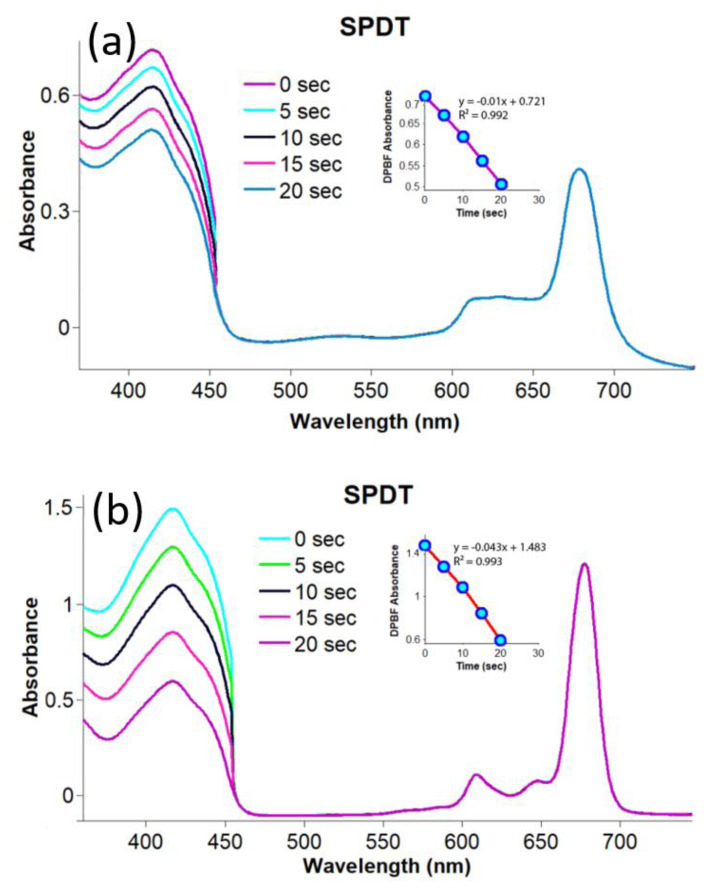
Representative absorption spectral changes during the determination of the singlet oxygen quantum yields for gold nanoconjugates **G-2** and **G-4** in DMSO by sonophotochemical applications; a) compound **G-2** (concentration: 4 × 10^−6^ M) and b) compound **G-4** (concentration: 10 × 10^−6^ M).

In the sonophotochemical studies, compounds **2**–**4** and gold nanoconjugates **G-2** and **G-4** were irradiated by both ultrasound and light for 10 s (firstly 5 s for ultrasound and then 5 s for light). The changes obtained are shown in Figures 7 and 8. The Φ_Δ_ values calculated for compounds **2**–**4** and gold nanoconjugates **G-2** and **G-4** are listed in the Table. Although the ultrasound frequency applied has been reported as approximately 1 MHz for SPDT applications in the literature [63,64], the frequency of ultrasound chosen was 35 kHz to evaluate the effect of ultrasound irradiation on the singlet oxygen production capacities of the photosensitizing candidates studied using the same UV-Vis spectra [65]. The Φ_Δ_ values of compounds **2**–**4** and gold nanoconjugates **G-2** and **G-4** increased significantly under sonophotochemical irradiation. The highest singlet oxygen quantum yield was obtained for compound **3** owing to the heavy metal effect of lutetium. Moreover, compound **4** exhibited much higher singlet oxygen quantum yield than silicon(IV) phthalocyanine dichloride (Φ_Δ_= 0.18 in DMSO) [66]. The result obtained confirmed the improving effect of the substituted thiazole group on singlet oxygen generation. Further, the singlet oxygen generation capacities of compounds **2** and **4** were improved significantly by the conjugation with gold nanoparticles. Indeed, metallic nanoparticles can increase the surface area for bubble nucleation and in turn enhance the efficiency of acoustic cavitation and ROS generation in US-mediated treatments [67–71]. Therefore, the singlet oxygen generation capacities of compounds **2**–**4** and gold nanoconjugates **G-2** and **G-4** were increased by applying the sonophotochemical methods even at low frequency. Karakılıç et al. synthesized new symmetric and asymmetric thiazolidin-4-one and/or 2-(4-(2-hydroxyethoxy)phenyl)-3-phenylthiazolidin-4-one containing zinc phthalocyanines and studied their photophysical and biological properties. The singlet oxygen quantum yields obtained were between 0.04 and 0.78. The symmetric zinc phthalocyanine exhibited the highest singlet oxygen production capacity [72]. Dilber et al. investigated the photophysical and photochemical features of new asymmetrically substituted metal-free and zinc phthalocyanine derivatives bearing 4-(4-(5-phenyl-1,3,4-oxadiazol-2-yl)phenoxy) and 4-(2-(benzo[d]thiazol-2-yl)phenoxy) groups. The singlet oxygen quantum yields obtained were 0.17 and 0.69 [73]. Nene and Nyokong prepared water soluble zinc phthalocyanine-bearing thiazole groups. The resultant compound was used for the surficial functionalization of gold and silver nanoparticles. The photosensitizing properties of the phthalocyanine-metal nanoconjugates were studied by performing light, ultrasonic, and light/ultrasonic activation methods. The singlet oxygen of the studied nanohybrids significantly increased with light/ultrasonic activation. Similar results were achieved for the studied compounds (**2**–**4**) and nanoconjugates (**G-2** and **G-4**) [74]. Additionally, the findings confirmed the improvement in the Φ_Δ_ values of phthalocyanines after linkage to the surface of gold nanoparticles [61,62].

### 3.4. Photodegradation quantum yield (Φ_d_)

**Figure 9:**
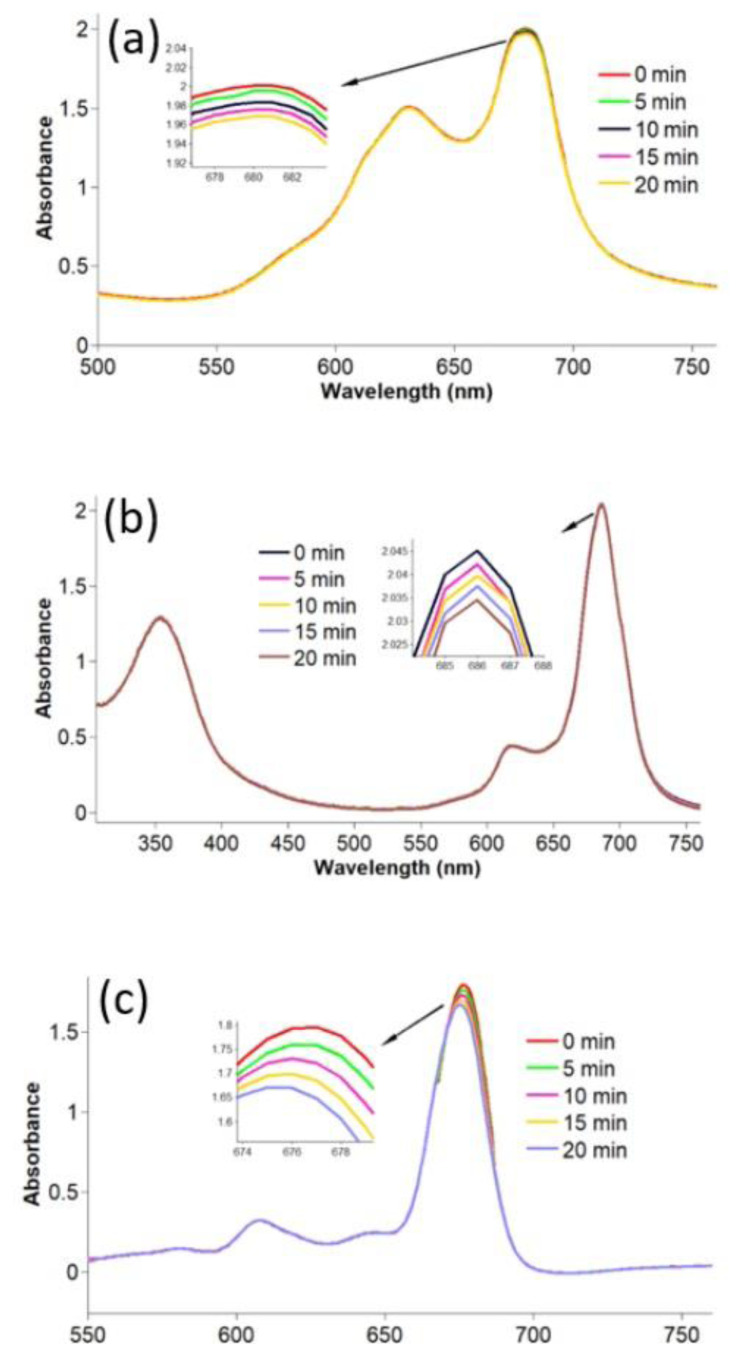
The spectrum of the determination of the photodegradation quantum yield of compounds **2**–**4** in DMSO; a) compound **2**, b) compound **3**, and c) compound **4** (concentration: 12 × 10^−6^ M).

**Figure 10:**
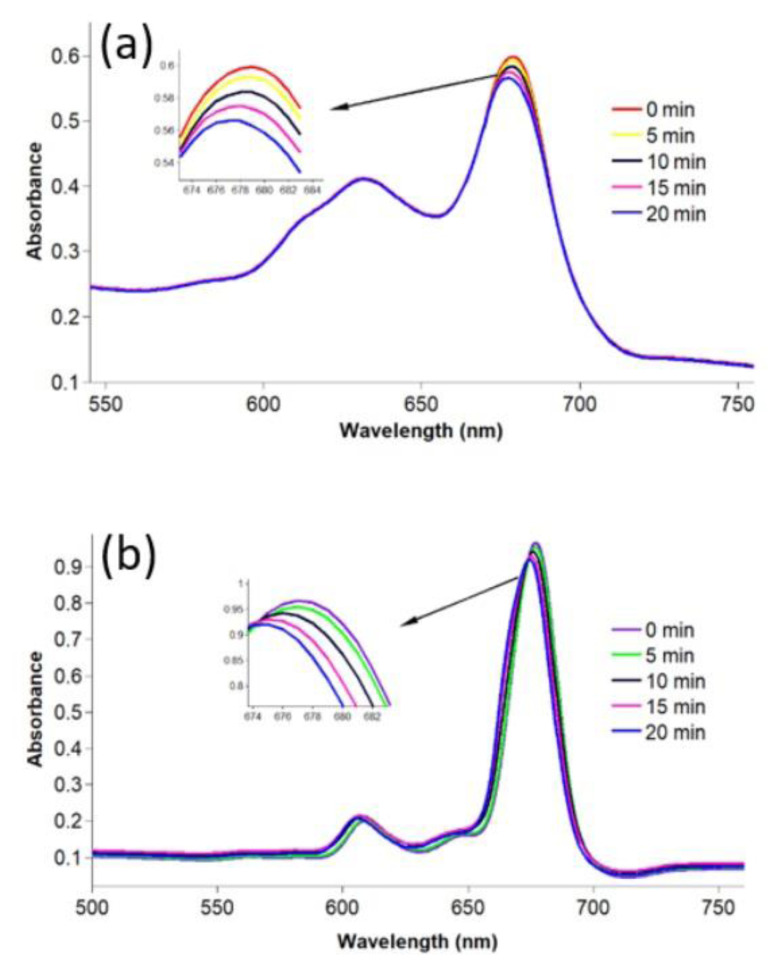
The spectrum of the determination of the photodegradation quantum yield of nanoconjugates **G-2** and **G-4** in DMSO; a) compound **G-2** and b) compound **G-4** (concentration: 12 × 10^−6^ M).

A suitable photosensitizer exhibits high stability under photoirradiation in PDT and SPDT applications. The Φ_d_ value is an efficient parameter to measure the stability of complexes. Accordingly, the photostability of a photosensitizer is usually investigated by studying the change in its UV-Vis spectrum during a certain period of photoirradiation. In the present study, the decreases in the intensity of the Q band maxima of compounds **2**–**4** and gold nanoconjugates **G-2** and **G-4** were examined after each 5-min photoirradiation (Figures 9 and 10). The Φ_d_ values are summarized in the Table. Compound **4** exhibited higher stability than compounds **2** and **3**. Additionally, the conjugation of compound **4** to the surface of gold nanoparticles slightly improved its photostability. Nas et al. studied the photodegradation quantum yields of benzothiazole-containing tetra-substituted metal-free and metal (Pb(II) and Zn(II)) phthalocyanines as well. The quantum yields obtained were between 0.09 and 3.43 × 10^−3^ [56]. Karakılıç et al. examined the photodegradation potential of symmetric and asymmetric zinc phthalocyanines bearing thiazolidin-4-one and 2-(4-(2-hydroxyethoxy)phenyl)-3-phenylthiazolidin-4-one groups. The quantum yields obtained were between 0.038 and 0.074 × 10^−3^ [71]. In comparison to the literature, the compounds displayed moderate stability and can be considered photosensitizer candidates [65,75].

## 4. Conclusion

In the present study, a series of peripherally or axially substituted metal phthalocyanines {M = Zn(II), Lu(III), Si(IV)} bearing chlorine and 2-(4-methylthiazol-5-yl) ethoxy groups were synthesized and characterized. Zinc(II) and silicon(IV) phthalocyanines were conjugated to the surface of gold nanoparticles. The photophysicochemical properties of all the resultant compounds and nanoconjugates were examined by photochemical and sonophotochemical methods. The linkage of phthalocyanines to the gold nanoparticles’ surface led to higher singlet oxygen quantum yields, which might have resulted from the conservation of their monomer structure. Furthermore, the presence of thiazole groups on the phthalocyanine ring or axial position improved the singlet oxygen generation. Although the highest singlet oxygen quantum yield was obtained for the lutetium(III) phthalocyanine, the substitution of a chlorine atom with a thiazole group in the silicon(IV) phthalocyanine dichloride significantly increased its singlet oxygen production capacity. In addition, the Φ_Δ_ values that were obtained by applying photochemical methods became approximately double by performing sonophotochemical methods.

## Supplementary Material

### Materials and equipment

Fluorescence spectra were measured with a Varian Eclipse spectrofluorometer using 1-cm path length cuvettes at room temperature. Photoirradiations were measured using a General Electric quartz line lamp (300 W). A 600-nm glass cut-off filter (Schott) and a water filter were used to filter off ultraviolet and infrared radiations, respectively. An interference filter (Intor, 700 nm with a bandwidth of 40 nm) was additionally placed in the light path before the sample. Light intensities were measured with a POWER MAX5100 (Mol electron detector incorporated) power meter. Bandelin Ultrasonic RK 100 H was used for ultrasound irradiation.

### Photophysical and photochemical studies

#### Fluorescence quantum yields (Φ_F_)

Fluorescence quantum yield (Φ_F_) was determined by applying the comparative method (Eq. 1) [1, 2],


(1) $$\Phi_{\text{F}}=\Phi_{\text{F}(\text{Std})}\frac{\text{F}.{\text{A}}_{\text{Std}}.{\text{n}}^{2}}{{\text{F}}_{\text{Std}}.\text{A}.{\text{n}}_{\text{Std}}^{2}}$$

Here F and F_Std_ are the areas under the fluorescence emission curves of the sample and the standard, respectively. A and A_Std_ are the respective absorbances of the samples and standard (unsubstituted **ZnPc**) at the excitation wavelengths, respectively. *n^2^* and *n^2^*_Std_ are there fractive indices of solvents used for the sample and standard, respectively. Unsubstituted **ZnPc** (Φ_F_ = 0.20 in DMSO) [2] was used as the standard. The samples and standard were excited at the same wavelength.

#### Singlet oxygen quantum yields (Φ_Δ_)

Singlet oxygen efficiency was determined in air (no oxygen bubbled) using the relative method (Eq. 2). Unsubstituted ZnPc was used as a reference, whereas 1,3-diphenylisobenzofuran (DPBF) was utilized as a chemical quencher for singlet oxygen,


(2) $$\Phi_{\Delta }=\Phi_{\Delta }^{\text{Std}}\frac{\text{R}.{\text{I}}_{\text{abs}}^{\text{Std}}}{{\text{R}}^{\text{Std}}.{\text{I}}_{\text{abs}}}$$

Here $\Phi_{\Delta }^{\text{Std}}$ is the singlet oxygen quantum yield for the standard ZnPc ( $\Phi_{\Delta }^{\text{Std}}=0.67$ in DMSO) [3]. R and R_Std_ are the DPBF photo bleaching rates in the presence of the samples and standard, respectively. I_abs_ and ${\text{I}}_{\text{abs}}^{\text{Std}}$ are the rates of light absorption by the sample and standard, respectively. The samples containing DPBF were prepared in the dark and irradiated in the Q band region. The absorption band of the DPBF was reduced by light irradiation (light intensity of 7.05 × 10^15^ photons s^−1^ cm^−2^). The degradation of DPBF was monitored using UV-Vis spectroscopy after each 5 s of light irradiation at 417 nm for PDT. For the sonophotochemical (SPDT) studies, the sample (the complex + DPBF) was monitored after each 10 s of irradiation (5 s by light intensity of 7.05 × 10^15^ photons s^−1^ cm^−2^ and 5 s by ultrasound at a frequency of 35 kHz).

#### Photodegradation quantum yields (Φ_d_)

Photodegradation quantum yields were determined using Eq. 3,


(3) $$\Phi_{\text{d}}=\frac{({\text{C}}_{0}-{\text{C}}_{\text{t}}).\text{V. }{\text{N}}_{\text{A}}}{\text{S.t. }{\text{I}}_{\text{abs}}}$$

Here C_0_ and C_t_ are the sample concentrations before and after irradiation, respectively, V is the reaction volume, N_A_ is the Avogadro’s constant, S is the irradiated cell area, t is the irradiation time, and I_abs_ is the overlap integral of the radiations of light intensity and the absorption of the sample. A light intensity of 7.05 × 10^15^ photons s^−1^ cm^−2^ was employed to determine the photodegradation [1]. The degradation of the Q band maximum was monitored after each 5 min of irradiation.

S1 FT-IR spectrum of compound **3**.

S2 ^1^H NMR spectrum of compound **3**.

S3 MALDI-TOF spectrum of compound **3**.

S4 FT-IR spectrum of compound **4**.

S5 ^1^H NMR spectrum of compound **4**.

S6 ^13^C NMR spectrum of compound **4**.

S7 MALDI-TOF spectrum of compound **4**.

Reference1
TayfuroğluÖ
KılıçarslanFA
AtmacaGY
ErdoğmuşA
Synthesis, characterization of new phthalocyanines and investigation of photophysical, photochemical properties and theoretical studiesJournal of Porphyrins and Phthalocyanines20182225026510.1142/S10884246185002812
AtmacaGY
Investigation of singlet oxygen efficiency of di-axially substituted silicon phthalocyanine with sono-photochemical and photochemical studiesPolyhedron202119311489410.1016/j.poly.2020.1148943
AtmacaGY
ErdoğmuşA
Synthesis of new water soluble silicon phthalocyanine substituted by linker sulfur atom and photophysicochemical studies for photodynamic therapyJournal of Porphyrins and Phthalocyanines2019231398140510.1142/S1088424619501487

## Figures and Tables

**Table:** Photophysical, photochemical and sonophotochemical properties of **2**–**4** and their gold nanconjugates in DMSO.

Biological candidate	Φ_F_	Excitation wavelength, nm	Φ_Δ (PDT)_	Φ_Δ (SPDT)_	Φ_d_ (10^−3^)
**2**	0.040	681	0.260	0.62	4.3
**3**	0.007	683	0.960	2.16	0.07
**4**	0.023	684	0.470	1.18	5.7
**G-2**	0.026	681	0.54	1.28	1.1
**G-4**	0.012	684	0.98	1.95	6.2
