# Supplementary figures and images for: Sonophotochemical and photochemical efficiency of thiazole-containing metal phthalocyanines and their gold nanoconjugates
Source: Turk J Chem. 2023 Sep 30;47(5):1085–102. doi: 10.55730/1300-0527.3596 (PMC10760820; doi:10.55730/1300-0527.3596)

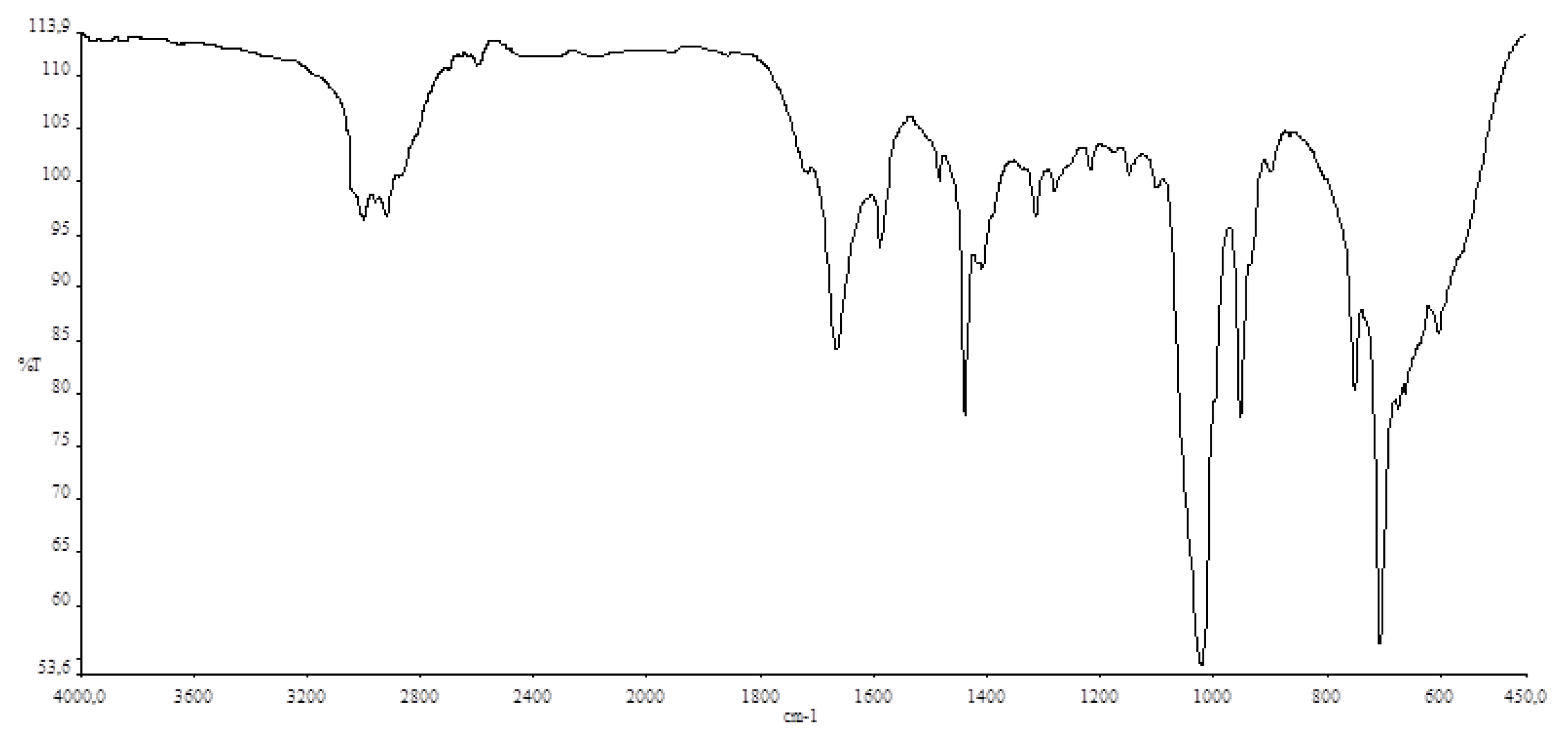

Supplement: S1 — FT-IR spectrum of compound 3. [file turkjchem-47-5-1085s1.tif]

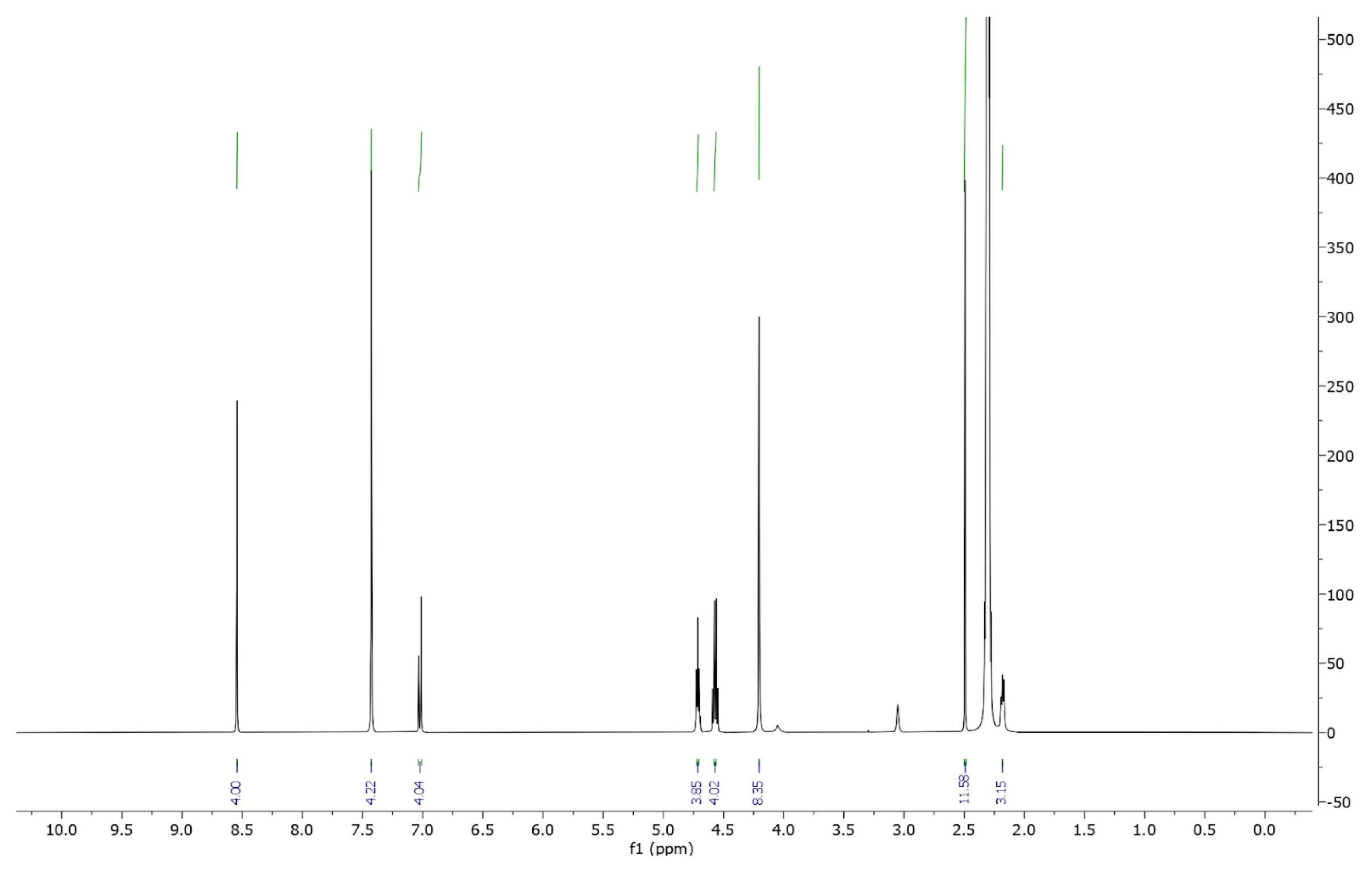

Supplement: S2 — 1H NMR spectrum of compound 3. [file turkjchem-47-5-1085s2.tif]

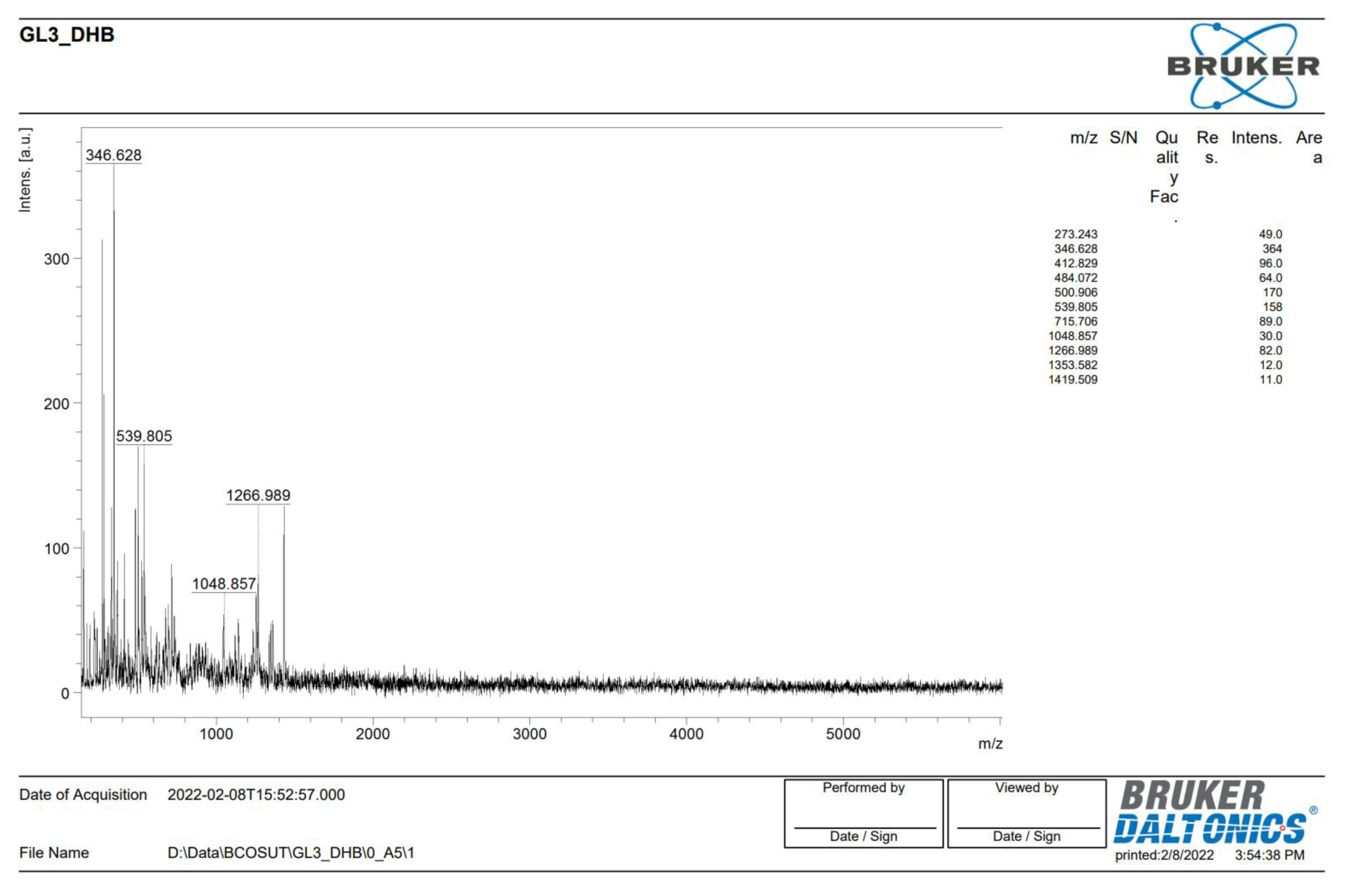

Supplement: S3 — MALDI-TOF spectrum of compound 3. [file turkjchem-47-5-1085s3.tif]

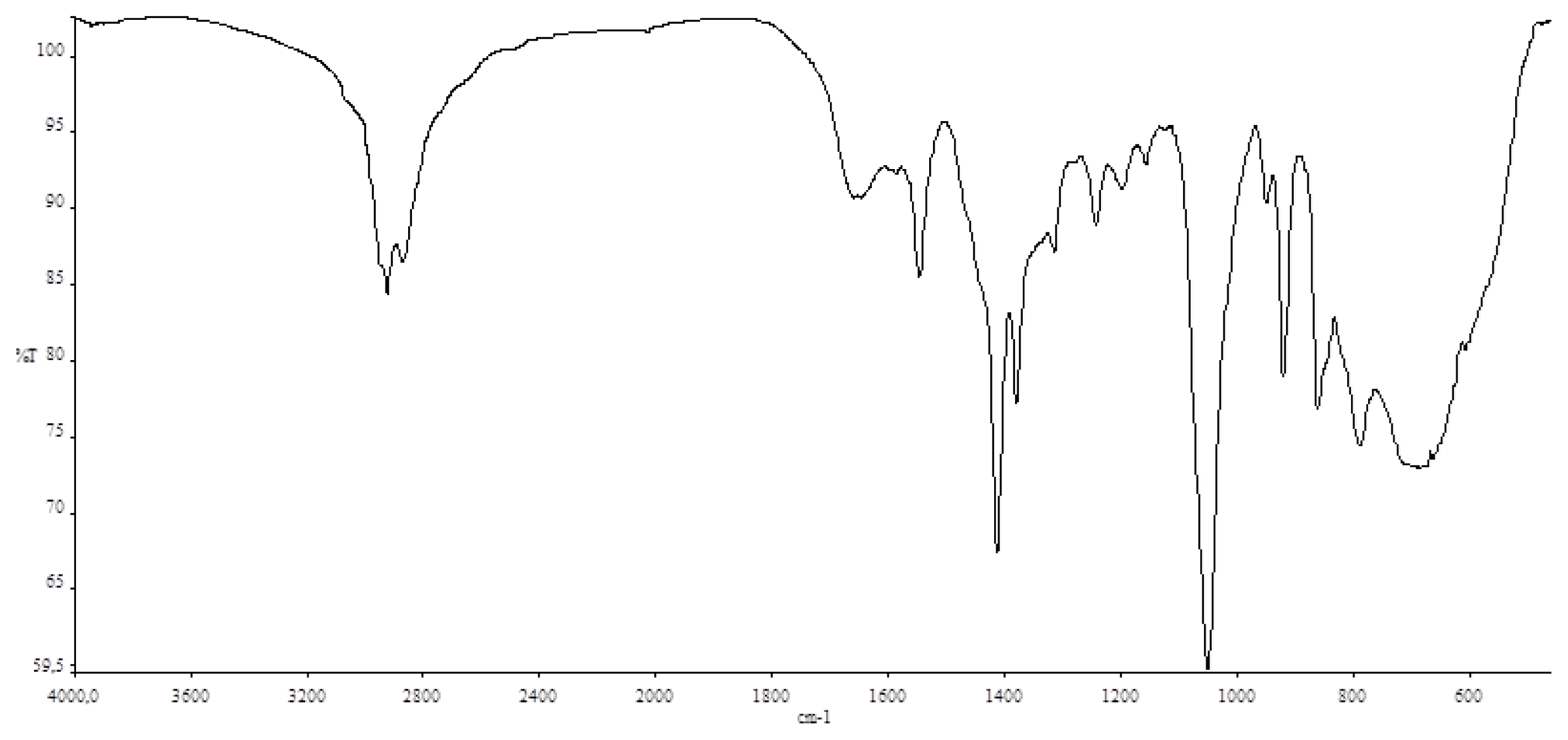

Supplement: S4 — FT-IR spectrum of compound 4. [file turkjchem-47-5-1085s4.tif]

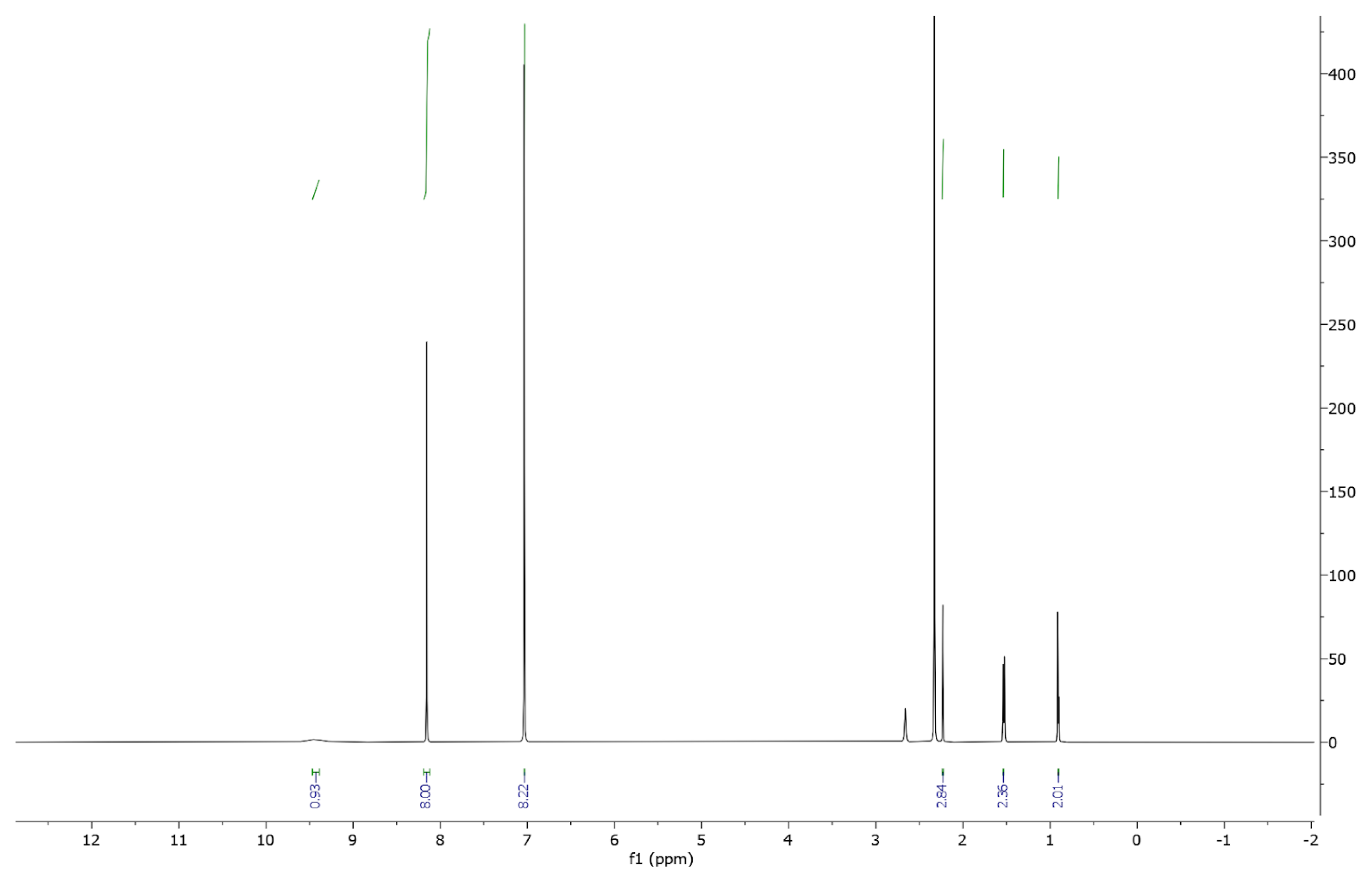

Supplement: S5 — 1H NMR spectrum of compound 4. [file turkjchem-47-5-1085s5.tif]

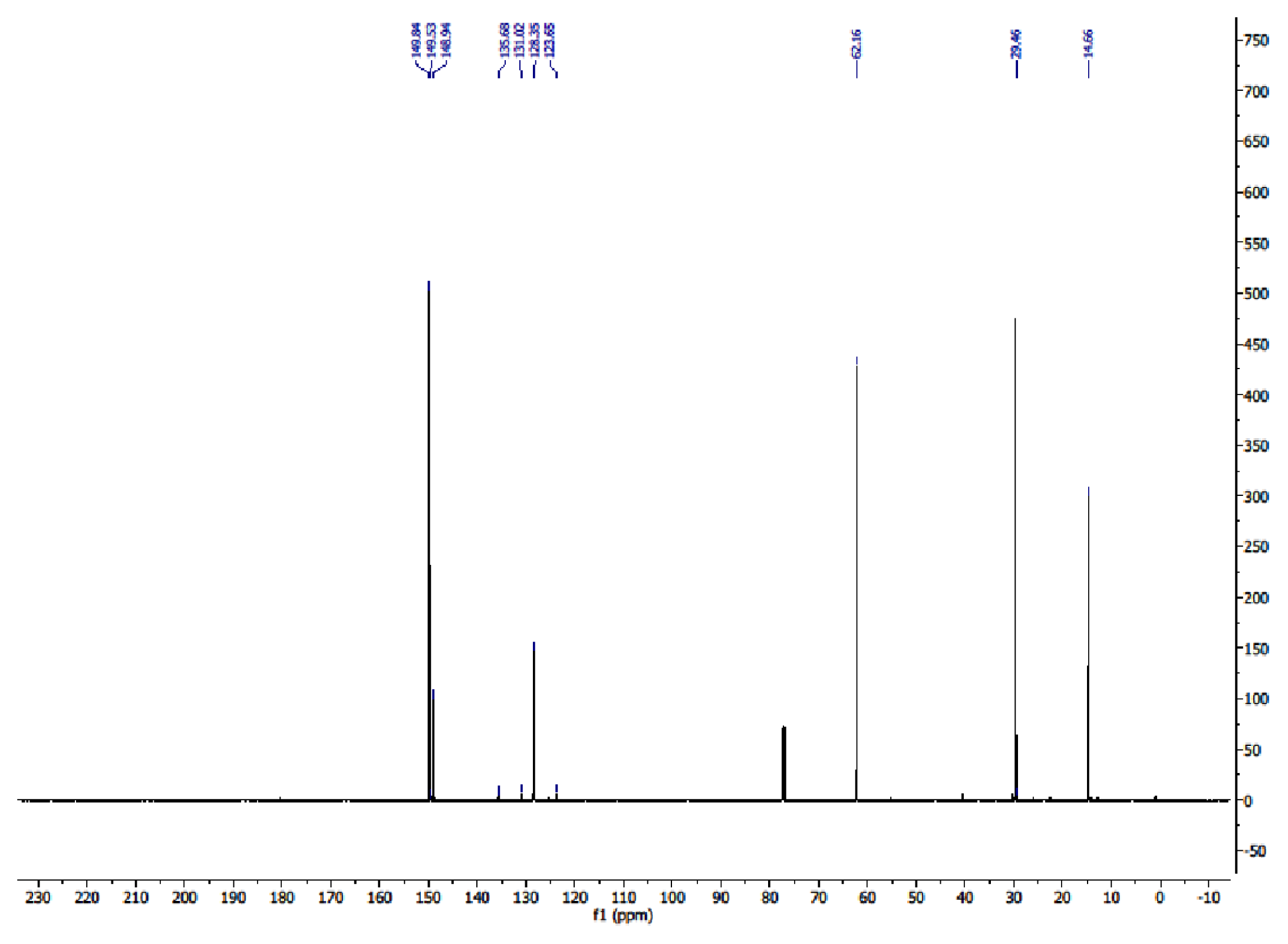

Supplement: S6 — 13C NMR spectrum of compound 4. [file turkjchem-47-5-1085s6.tif]

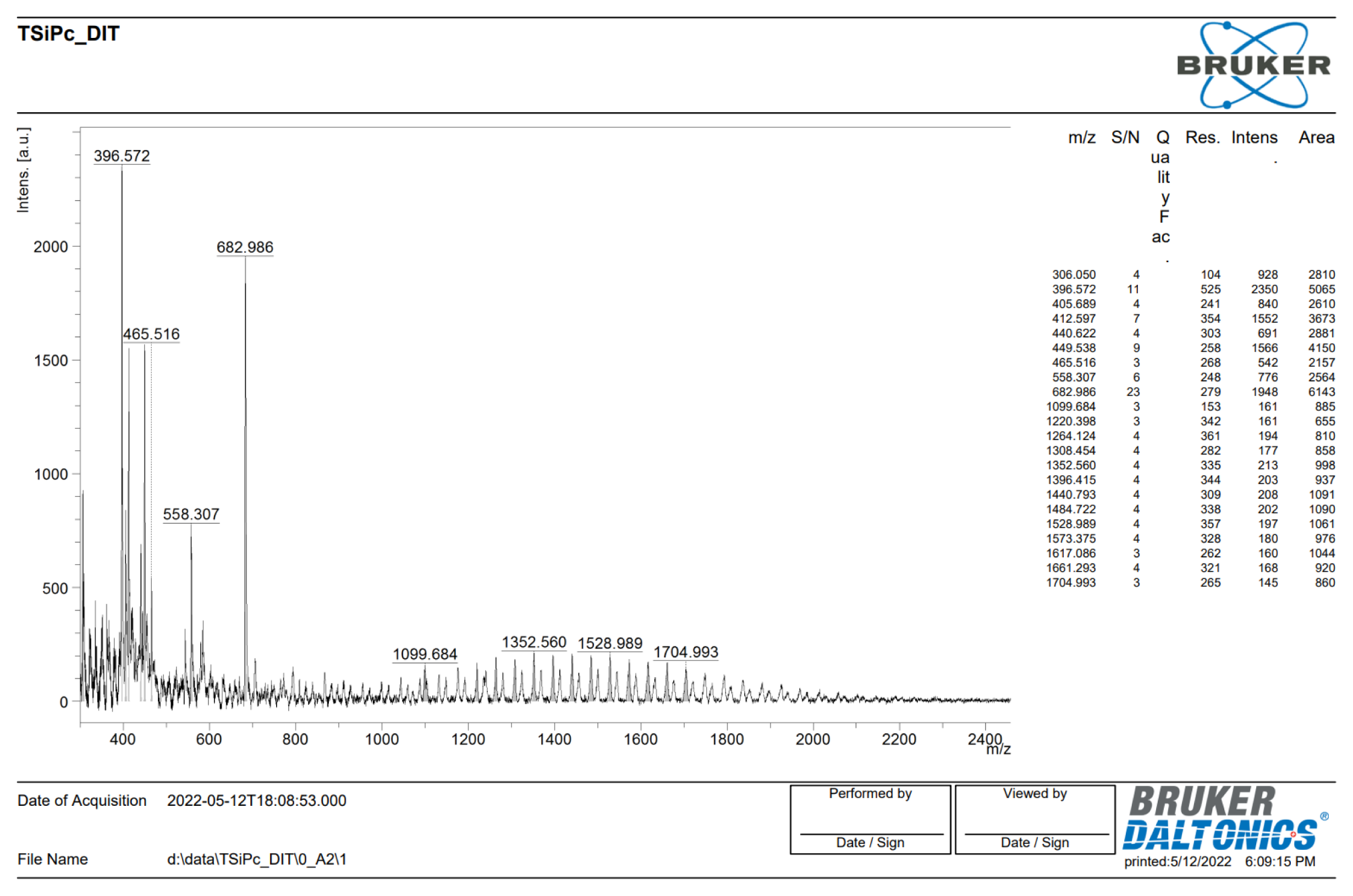

Supplement: S7 — MALDI-TOF spectrum of compound 4. [file turkjchem-47-5-1085s7.tif]
